# Numerical Simulation of Temperature Characteristics and Graphitization Mechanism of Diamond in Laser Powder Bed Fusion

**DOI:** 10.3390/ma16186338

**Published:** 2023-09-21

**Authors:** Yongqian Chen, Shanghua Zhang, Jialin Liu, Wei Zhang, Qingyuan Ma, Xiwang Wu, Shirui Guo, Yinghao Cui, Xiaolei Li, Bo Zheng, Lujun Cui

**Affiliations:** 1 School of Mechatronics Engineering, Zhongyuan University of Technology, Zhengzhou 451191, China; 2 Zhengzhou Key Laboratory of Laser Additive Manufacturing Technology, Zhengzhou 451191, China; 3 Powder Metallurgy Research Institute, Central South University, Changsha 410083, China; 4 Henan Huanghe Whirlwind Co., Ltd., Xuchang 461500, China

**Keywords:** laser powder bed fusion, diamond tools, laser processing

## Abstract

Thermal damage to diamonds is a major limitation in laser powder bed fusion (LPBF) processing of metal matrix diamond composites. In this paper, a numerical simulation model was established to describe the thermal effect of the Diamond-CuSn10 composite on the LPBF process. The simulation results show that the temperature of the diamond presents a double-peak structure, and the double-peak temperature curve shape can be modulated by modifying the laser scanning offset and the size of the diamond powder. And it suggests that the heat of the diamond mainly comes from the transfer of the molten pool. Then, combined with the experimental phenomenon, the mechanism of diamond graphitization in the LPBF process is analyzed. It indicates that since the surface defects of the diamond inhibit the heat conduction of the diamond, the temperature accumulates on the surface, leading to the graphitization of the diamond. Finally, based on this model, the potential of Ti-coated diamonds to prevent and reduce thermal damage in the LPBF process has been extensively studied. It is found that a Ti coating with low thermal conductivity can effectively reduce diamond temperature and improve diamond graphitization resistance. This study can provide a good method and basis for the preliminary selection of LPBF process parameters and the understanding of the graphitization mechanism of diamond tools.

## 1. Introduction

Diamonds are promising for their high hardness, wear resistance, and chemical stability. They have been widely applied in the field of precision manufacturing [1,2,3,4]. As an advanced diamond tool manufacturing technology in recent years, laser powder bed fusion (LPBF) has drawn much attention and has been studied [5,6]. In the field of diamond tool fabrication through LPBF, the extent of the thermal damage to the diamonds is a crucial indicator that determines whether the diamond retains its excellent performance after processing, directly impacting the wear resistance and service life of diamond tools.

Diamonds are at risk of damage under laser irradiation. Damage types can be divided into light-induced damage and thermal-induced damage. When the laser power density is extremely high (up to a few GW), it’s enough to dissociate C-C covalent bonds in a diamond lattice. The light-induced graphitization occurs by electrons absorbing energy through an inverse bremsstrahlung process and, thus, facilitating a transition from an sp3 tetrahedral to an sp2 aromatic and/or an sp2 olefinic bonding state. On the other hand, in the LPBF process field, the continued (or long pulse width) laser will be absorbed by the diamond and metal powder, and the heat generated in this process will generate a thermal graphitization effect on the diamond.

In addition to graphitization damage, thermal-induced cracks are also important diamond damage in LPBF, and its resistance to damage depends on the diamond grade and the contact medium surrounding the diamond [7]. Thermal-cracking damage is caused by the rapid temperature rise of the diamond and the different thermal expansion coefficients between the diamond and the matrix. Large temperature gradients and fluctuations lead the diamond to crack or even explode, and exacerbate the stress between the diamond and the matrix [8]. During process optimization, it is crucial to avoid significant temperature gradients and fluctuations.

As mentioned above, diamond temperature is very important in LPBF processing. The phase-transition temperature of diamond varies in different media, with graphitization starting at 850 °C in air, 1200 °C in a vacuum environment, and 1500 °C in inert gases [9]. By using a coated diamond, the damage resistance of the diamond can be improved and the wettability of the diamond matrix can be improved. However, it is challenging to measure the temperature of diamonds directly during the processing, often requiring simulation methods to obtain the diamond temperature. Y. Wang et al. established a three-dimensional Finite Element (FE) model to numerically analyze the temperature parameters of diamond grain during pulse laser spot heating for brazing [10]. J. Gan et al. established a temperature field model for single-track scanning with different laser powers, resulting in appropriate SLM process parameters [11]. Z. Zhou et al. constructed a finite element analysis temperature field model for multi-track scanning in laser powder bed fusion with a mixed powder bed, based on ray tracing theory [12]. In the aforementioned studies, the temperature of the diamond is ascertained through two distinct methods: either by direct measurement of the molten pool temperature or by employing the overall absorption rate subsequent to powder blending as a simulation parameter. While this model aptly elucidates the temperature field distribution within the LBPF process, it regrettably falls short in encapsulating the exceptional characteristics inherent to diamonds when juxtaposed against alloy powder within the model’s purview.

In our previous work, a single-channel scanning model of the temperature field was established by precisely regulating the laser absorption of powder at different locations, and a single display of diamond temperature was realized. By combining simulation results and the diamond graphitization phenomenon, we successfully replicated the thermal damage process of diamond abrasives in the Powder Bed Fusion-Laser Beam (PBF-LB) process and experimentally verified the temperature threshold for thermal damage transformation. Furthermore, a quantitative relationship model, “PBF-LB process-abrasive temperature-graphitization degree-mechanical performance”, was established [13]. In this paper, we delve deeper into the thermodynamic behavior of diamonds within the molten pool. The double-peak structure of the diamond temperature shows that the diamond temperature mainly comes from the heat transfer of the molten pool. Combined with the experimental data, the graphitization mechanism of diamond was analyzed. In addition, by changing the deviation of the laser scanning path and the diamond diameter, the basic control of the bimodal structure is realized. Based on this model, the potential of diamond coating to prevent and reduce thermal damage in the LPBF process has been extensively studied. It is found that a Ti coating with low thermal conductivity can effectively reduce diamond temperature and improve diamond graphitization resistance. The research results can provide a good method and basis for the preliminary selection of the LPBF process parameters and the understanding of the graphitization mechanism of diamond tools.

## 2. Materials and Methods

### 2.1. Sample Preparation

**Figure 1:**
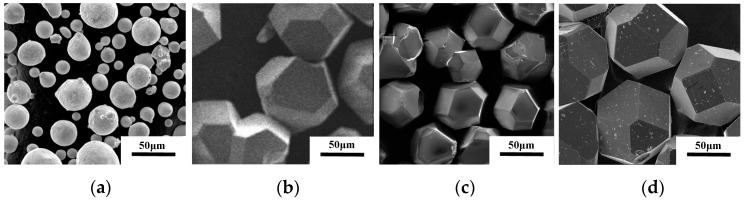
SEM images of different powders (**a**) CuSn10 alloy powder, uncoated diamond particles (**b**) 74 μm~90 μm (**c**) 45 μm~53 μm, (**d**) Ti-coated diamond particles (78 μm~95 μm).

Gas atomized CuSn10 alloy powder (15 μm~53 μm), diamond particles (HFD-D, 45 μm~53 μm, and 74 μm~90 μm) and magnetron sputtered Ti-coated diamond particles (78 μm~95 μm) were employed as raw materials, as shown in Figure 1. The CuSn10 alloy powder, shown in Figure 1a, exhibits favorable sphericity and fluidity, facilitating the molten flow process. The uncoated diamond particles with sizes of 74 μm~90 μm and 45 μm~53 μm are shown in Figure 1b,c, respectively. Figure 1d shows the morphology of magnetron sputtered Ti-coated diamond particles with high purity Ti (99.999%) target material, and a coated temperature of 300–400 °C. Finally, the formed Ti coating is dense and uniform.

### 2.2. Laser Powder Bed Fusion Methods

**Figure 2:**
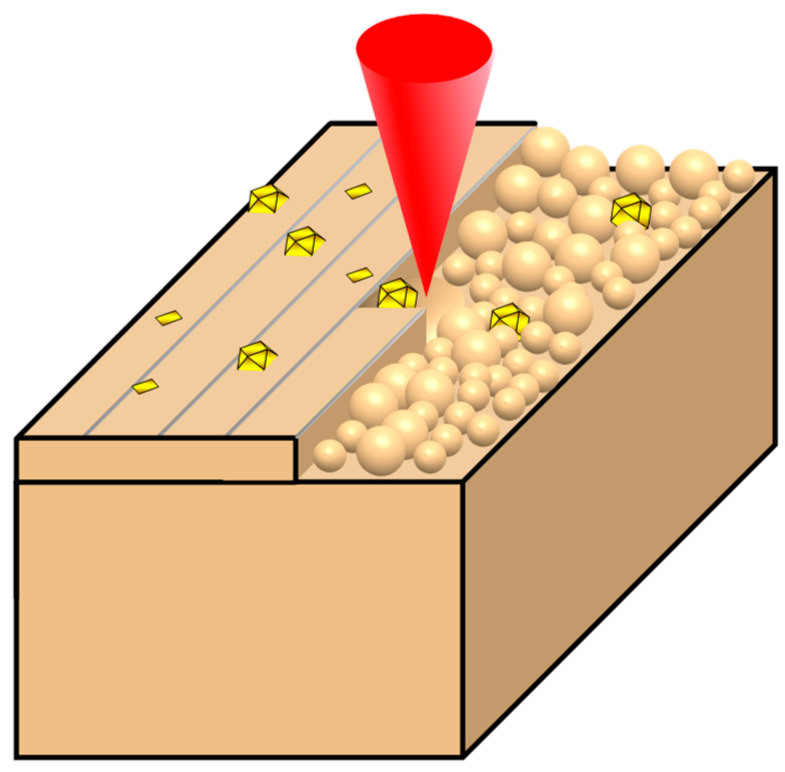
Schematic of the CuSn10-Diamond metal matrix diamond composites by LPBF process.

The alloy powder and diamond particles were uniformly mixed using a 3D mixer (Zhengzhou Hejin Powder Technology, Zhengzhou, China) for 5 h, and the diamond concentration was 12.5 vol%. The mixed powders were dried at 80 °C for 24 h. The CuSn10-Diamond composite samples were fabricated utilizing SLM equipment (WXL-120E, Xiamen Wuxinglong Technology Co., Ltd., Xiamen, China). The equipment consists of a CW laser with a laser wavelength of 1064 nm, a maximum power of 500 W, and a spot diameter of 50 μm, with Gaussian energy distribution. The composite was fabricated on a pure copper build-up substrate (116 mm × 116 mm × 20 mm). During the processing, the temperature of the substrate was maintained at about 100 °C and the oxygen content in the bench was ≤400 ppm. The specific process parameters are shown in Table 1, and the schematic of the CuSn10-Diamond metal matrix diamond composites by LPBF is shown in Figure 2.

### 2.3. Microstructures and Properties Characterization

Scanning electron microscopy (SEM) with back-scattering mode (FEI Quanta FEG 250, Eindhoven, The Netherlands) was used to analyze the original morphology of the raw materials and the microstructure of the samples. The graphitization of the diamonds was analyzed by the Renishaw in Via Raman microscope system with a 532 nm wavelength and a 500–2000 Raman shift/cm^−1^ of detection range.

## 3. Numerical Simulation Model of LPBF Process

### 3.1. Governing Equations

The 3D heat transfer equations can be described as [14]:

(1) $$\rho c\frac{\partial T}{\partial t}=\frac{\partial }{\partial x}\left(k_{x}\frac{\partial T}{\partial x}\right)+\frac{\partial }{\partial y}\left(k_{y}\frac{\partial T}{\partial y}\right)+\frac{\partial }{\partial z}\left(k_{z}\frac{\partial T}{\partial z}\right)+Q,$$

where *ρ* is the material density (kg/m^3^), *c* is the specific heat (J/(kg·°C)), *T* is the current temperature (°C), *t* is the time (s), *x*, *y*, and *z* are the coordinates in the reference system (m), *k_x_*, *k_y_*, and *k_z_* are the thermal conductivity (W/(m·°C)) of *x*, *y*, and *z*-axis direction, and *Q* is the inner heat source (W/m^3^).

The environment of the LPBF process has a constant temperature (room temperature). For the initial conditions:

(2) $$T(x,y,z,0)=T_{0},$$

where *T*_0_ is the ambient temperature at 22 °C.

The energy input of the laser is the second boundary condition. In this paper, the Gaussian heat source model was used in the simulation, and the radius of the Gaussian spot in this paper is defined by the “1/e^2^ energy method”. Based on this assumption, the expressions of the Gaussian surface heat source model can be described as [13,14]:

(3) $$Q=\frac{2\eta P}{\pi r_{0}{}^{2}}\exp\left[-2{\left(\frac{x^{2}+(y-vt^{2})}{r_{0}}\right)}^{2}\right],$$

where *P* is the average power of the laser, *r*_0_ is the radius of the laser beam, *η* is the absorption rate, and *v* is laser scan velocity.

Convection heat transfer and radiation heat transfer are boundary conditions and the main ways of heat loss. In this model, the thermal convection and radiation between the material and the air are considered, and the chemical reaction and molten pool flow are ignored. In addition, the heat transfer of the fine powder on the surface of the diamond to the diamond is ignored. The coupling formula can be expressed as [14]:

(4) $$-\vec{n}\cdot \left(-k\nabla T\right)=h\cdot \left(T_{ext}-T\right)+\varepsilon \sigma \left(T_{ext}{}^{4}-T^{4}\right),$$

where is the direction vector, *k* is the thermal conductivity, *h* is the convective heat transfer coefficient, *ε* is the emissivity of the powder bed, and *σ* is the Stefan-Boltzmann constant for radiation.

### 3.2. Numerical Simulation Model Establishment

**Figure 3:**
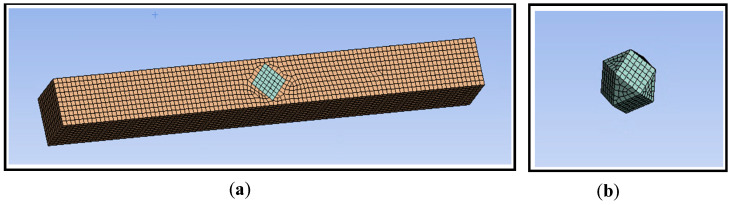
Mesh model (**a**) powder layer with (**b**) Diamond.

In order to investigate the thermal damage mechanism of diamond, an LPBF CuSn10-Diamond composite temperature field model was built using ANSYS^®^ finite element software 2020R2. The model consists of a powder layer and a diamond, and the structure is shown in Figure 3. The powder layer is CuSn10 pre-alloyed powder is 4000 μm × 1000 μm × 100 μm in size, and the diamond particle model is an 80 μm × 80 μm × 80 μm cube with 8 corners removed. The diamond particle model is embedded in the powder bed model. They are all meshed using regular hexahedrons with a mesh volume of 10 μm. The powder layer width was expanded when building the laser scanning path offset powder bed model in Section 4.2. In building the coated diamond powder bed model, a 2 μm titanium layer mated with diamond was placed on the diamond surface and embedded in the powder bed model in Section 4.4. Hexahedral mesh elements were used to discretize the computational domain. The grid side length is 10 μm. A time step of 5 μs was used in all simulations.

**Figure 4:**
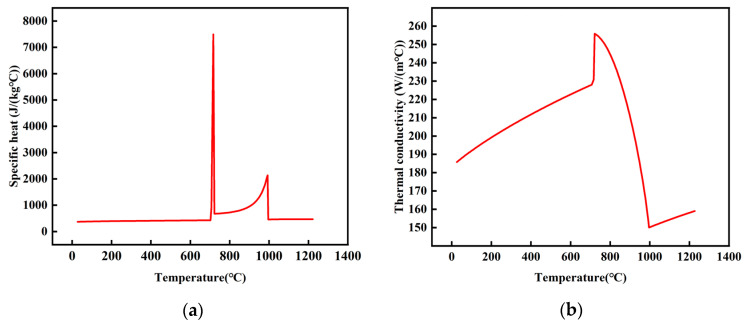
Material thermophysical parameter (**a**) Specific heat of the CuSn10 power (**b**) Thermal conductivity of the CuSn10 power.

Table 2 describes the thermophysical parameters of CuSn10, diamond, and titanium which are used in the simulation. The diamond and titanium in the model had been clearly defined in the ANSYS^®^ software. It is assumed that the adopted CuSn10 alloy is isotropic. The properties of the CuSn10, including thermal conductivity and specific heat, were measured using JMatPro^®^ software ver7, as shown in Figure 4a,b.

### 3.3. Hypothesis

(1) In the actual LPBF process, the laser is used as an external continuous-heating heat source, assuming that the energy of the heat source is a Gaussian distribution.
(2) Only consider the heat convection and heat radiation between the material and the air, ignoring the latent heat of the phase transition and other factors.
(3) Assume that the material is isotropic.
(4) It is assumed that the thermal conductivity of the diamond does not change with temperature.

## 4. Results

### 4.1. Thermodynamics of the Diamond

**Figure 5:**
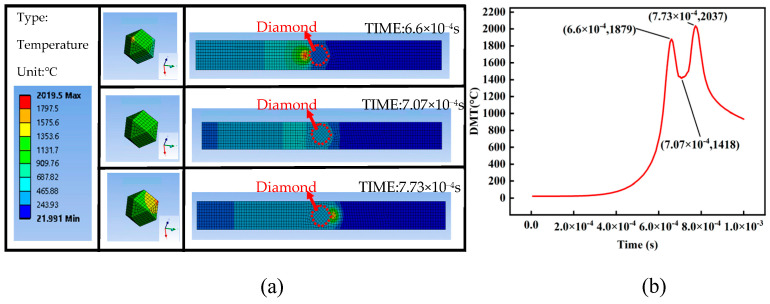
“Double-Peak” temperature field model (**a**) Temperature distribution cloud of the model at different times (**b**) DMT curve.

Figure 5 shows the simulation results, using the model established in Section 3 and the parameters in Table 1. Figure 5a is the temperature distribution cloud of the model at different times, and Figure 5b is the diamond’s maximum temperature (DMT) curve. In the LPBF process, the temperature of each part of the diamond at the same time is different. The DMT curve reflects the trend of the maximum temperature of the diamond changing with time during laser single-track scanning. It reveals the temperature change process of diamonds under the action of a laser and provides theoretical support for the analysis of the diamond graphitization temperature and the graphitization process.

As shown in Figure 5b, the DMT curve exhibits a “double-peak” structure. The double temperature peaks occurred in a very short period before and after the laser spot center illuminates the diamond, and the DMT curve valley occurred when the spot center irradiates the diamond. This interesting structure implied that the temperature of the diamond may mainly come from the heat transfer of the molten pool, rather than the laser irradiation.

**Figure 6:**
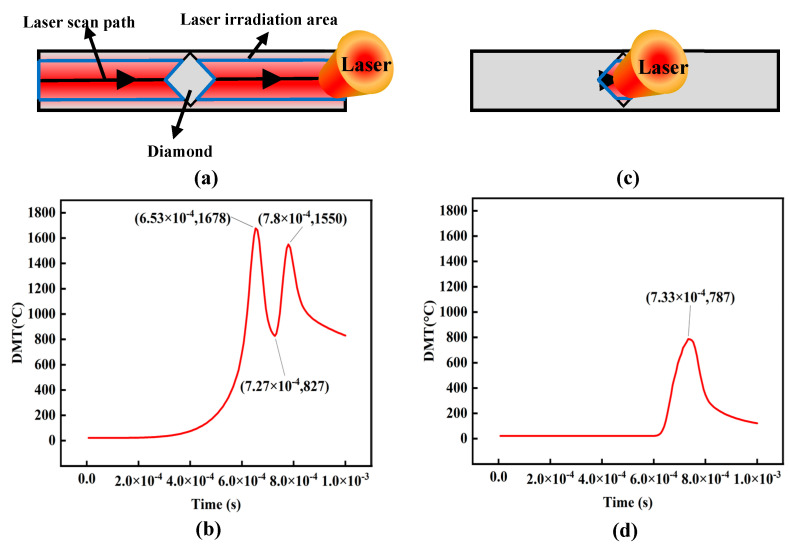
(**a**) Schematic diagram of Model I (**b**) DMT curve when laser irradiation solely on the CuSn10 (**c**) Schematic diagram of Model I (**d**) DMT curve when laser irradiation solely on the diamond.

To further study the major factors influencing diamond temperature, two distinct models were constructed as depicted in Figure 6a,c, respectively. In Model I, as shown in Figure 6a, the laser exclusively irradiates the CuSn10 during the LPBF process. Conversely, in Model II, as shown in Figure 6b, the laser exclusively irradiates the diamond during the LPBF process. The resulting DMT curves for these models can be observed in Figure 6b,d.

As shown in Figure 6b, the DMT curve reveals a distinctive “double-peak” structure that aligns with the typical model’s “double-peak” time node (as seen in Figure 5b). However, there is a notable difference between Figure 5b and Figure 6b: the post-peak temperature is lower than the front-peak in Figure 6b, while Figure 5 is the opposite. This shift is attributed to the absence of laser irradiation on the diamond, thereby impacting the accumulation of diamond temperature. On the other hand, as shown in Figure 6d, in Model II, the DMT curve demonstrates a single-peak structure, with a maximum DMT of 787 °C during the LPBF process, diverging from the outcome depicted in Figure 5b. Analyzing the peak shape and diamond temperature, it becomes evident that heat transfer from the molten pool holds greater significance than laser irradiation directly affecting the diamond. Therefore, it is inferred that the primary factor influencing the DMT lies in the heat transfer from the molten pool generated by laser irradiation on the CuSn10, rather than the direct transfer of laser energy to the diamond. Thus, thermal damage to the diamond primarily stems from heat transfer originating from the molten pool, while the direct energy transfer from the laser to the diamond assumes a subordinate role. In addition, the lowest ablation threshold of the diamond is approximately 1.21 × 10^7^ W/cm^2^ under direct laser irradiation at a wavelength of 1064 nm, and the maximum equivalent laser flux in this experiment is 4.3 × 10^6^ W/cm^2^, which is much lower than the diamond ablation threshold, which further proves the significance of the influence of heat transfer in the molten pool on the temperature of the diamond [13].

**Figure 7:**
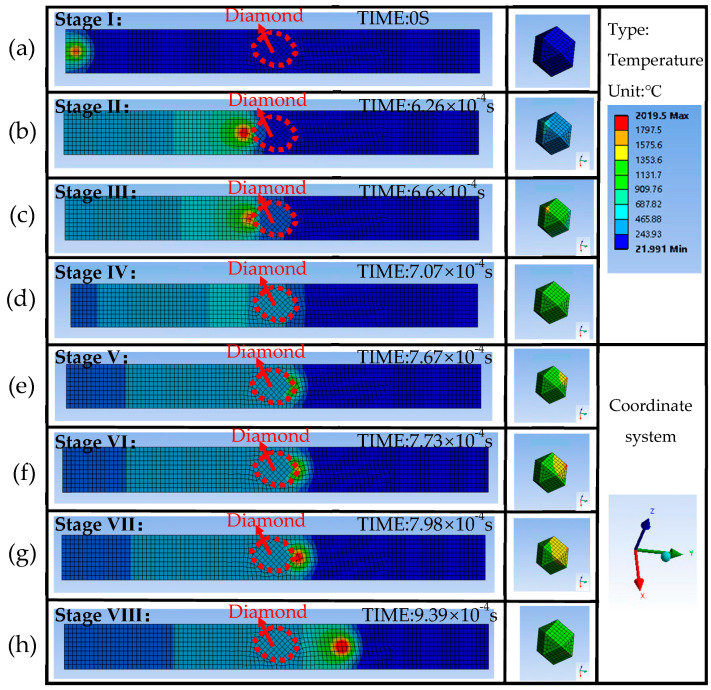
The process of molten pool movement and diamond temperature change.

Combining with Models I and II, the temperature distribution cloud of the diamond in the molten pool is studied. In our previous work [13], the process of diamond temperature variation in the molten pool is divided into three stages, which have been further refined in this paper. The specific movement of the molten pool and the corresponding diamond temperature variation can be divided into eight stages as shown in Figure 7.

- Stage I: As shown in Figure 7a, laser irradiation of the CuSn10 powder forms a molten pool. The molten pool is far from the diamond, and the diamond is still at room temperature.
- Stage II: As shown in Figure 7b, when the molten pool approaches, the heat of the molten pool is transferred to the diamond. In this stage, the heat is transferred from the molten pool through the unmelted metal powder to the diamond. The diamond heats up slowly.
- Stage III: As shown in Figure 7c, the diamond is in contact with the molten pool. The heat is transferred from the molten pool to the diamond directly, and the diamond heats up rapidly. The high thermal conductivity of the diamond makes it a micro heat dissipation channel. At the same time, the low absorption coefficient of the diamond weakens the laser energy intake, which causes the temperature of the molten pool to start decreasing. Due to the Gaussian energy distribution of a laser spot in space causing the center of the molten pool to have a higher temperature than the surrounding temperature, the DMT is affected by its position relative to the center of the molten pool, and the diamond temperature is highest when the center of the spot sweeps over the edge of the diamond.
- Stage IV: As shown in Figure 7d, the diamond is located in the center of the molten pool, and the low absorption coefficient of the diamond to the laser greatly reduces the heat intake of the melted powder. As a result, the temperature of the molten pool decreases rapidly, causing a corresponding decrease in the diamond’s temperature. When the laser beam is fully contained within the diamond, it experiences the least energy absorption, resulting in the lowest temperature for the molten pool. As a consequence, the diamond itself reaches its lowest temperature.
- Stage V: As shown in Figure 7e, with the movement of the laser spot, the part of the laser that irradiates the CuSn10 is increased. Laser energy intake is increased, the molten pool temperature increases, and the diamond temperature is back in the rising state.
- Stage VI: As shown in Figure 7f, the center of the laser irradiates the CuSn10 again. The high energy density raises the temperature of the molten pool, and the diamond temperature reaches its maximum.
- Stage VII: As shown in Figure 7g, the center of the laser is gradually moving away from the diamond, but the diamond is still in the molten pool. As the diamond moves away from the center of the spot, the diamond cools down rapidly.
- Stage VIII: As shown in Figure 7h, the molten pool solidifies, the diamond is embedded in the body, and the diamond dissipates slowly with the temperature of the body.

**Figure 8:**
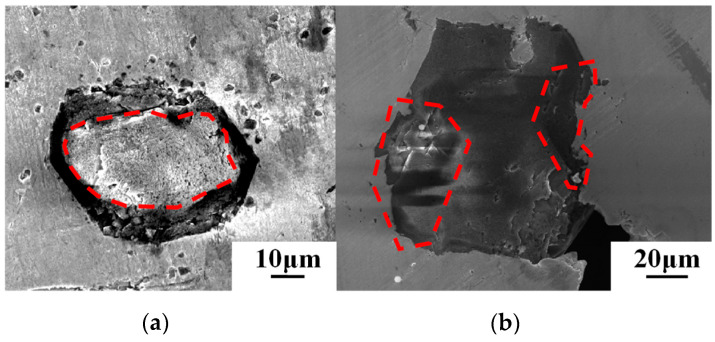
SEM image of the diamond particle (**a**) Serious thermal damage on the diamond with a diameter of 45 μm~53 μm (**b**) Slight thermal damage on the diamond with a diameter of 74 μm~90 μm.

In order to verify the accuracy of the simulation conclusions, the SEM image of the diamond particle profile where the thermal damage occurred was analyzed, as shown in Figure 8a,b. The two samples were manufactured by SLM equipment using diamond particles with the laser parameters in Table 1.

Figure 8a represents a diamond sample that has undergone serious thermal damage. It shows that the region where the matrix is in contact with the diamond (outside the dashed line) exhibits more pronounced thermal damage in contrast to the central portion unaffected by the matrix (inside the dashed line). A distinct graphite layer forms between the diamond and the matrix, aligning with the findings presented in the literature [7], which identified the formation of an 8 μm graphite layer surrounding the diamond. Therefore, the observation leads to the conclusion that, in the LPBF process, thermal damage of the diamond primarily manifests on its surface.

Diamond possesses exceptionally high thermal conductivity, facilitating rapid temperature propagation throughout its structure. Therefore, every region of the diamond should undergo thermal damage simultaneously. However, as depicted in Figure 8b, the diamond sample exhibits slight thermal damage, with only two areas of thermal damage marked by a red dash. It implied that the temperature does not accumulate within the diamond due to its high thermal conductivity. However, the defect decreases the local thermal conductivity, causing temperature accumulation specifically at these defect sites, consequently leading to thermal damage to the diamond.

**Figure 9:**
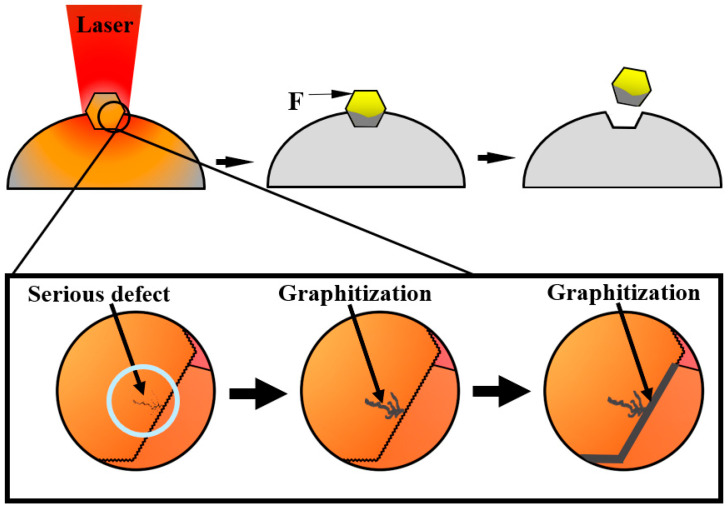
Diamond thermal damage process and diamond shedding by force.

Figure 9 illustrates the thermal damage process of the diamond powder. The original defect exists in each diamond micro-particle, and its damage temperature threshold is much lower than the pure diamond. According to the simulation results, the temperature of the diamond primarily stems from heat conduction originating from the molten pool. As shown in Figure 9, when the defect contacts the molten pool, it heats up until it reaches the threshold and then begins the graphitization process; when the molten pool continues to transfer energy to the diamond, the graphitization range enlarges as the temperature increases, leading to serious thermal damage, as in Figure 8a,b. Moreover, because the defects often appear on the diamond surface, when the graphitization becomes very serious, the binding force between the matrix and the diamond decreases. When the diamond is subjected to horizontal force, the graphitized diamond is easy to fall off, resulting in a low utilization rate of the diamond, shortened service life of diamond tools, reduced wear resistance, and poor processing quality [9].

### 4.2. The Effect of Laser Scan Path Offset on the Diamond Temperature

**Figure 10:**
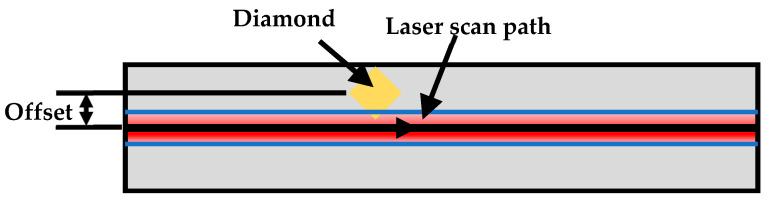
Diagram of the offset between the laser scanning path and the diamond.

Based on the “double-peak” model, the laser-diamond offset has an impact on the DMT curves, consequently influencing the quality of the manufacturing process. In order to study this relationship, this section focuses on the analysis of diamond temperature changes relative to different positions of the molten pool. This is achieved by intentionally offsetting the relative position of the laser scanning path and the diamond, as illustrated in Figure 10.

In the powder-laying method employed in LPBF, the positioning of diamonds may occur randomly, leading to variations in the trajectory relative to the laser to the diamond. Additionally, when the diamond is within the molten pool, it is subject to the combined effects of Marangoni convection and gravity. These factors contribute to the diamond being positioned above the molten pool and subsequently migrating toward the sides, resulting in potential deviations from the intended scanning path [17,18]. Therefore, the possible presence of powders on the diamond surface was ignored and the effect of the offset between the laser scanning path and the diamond on the DMT curve was studied.

**Figure 11:**
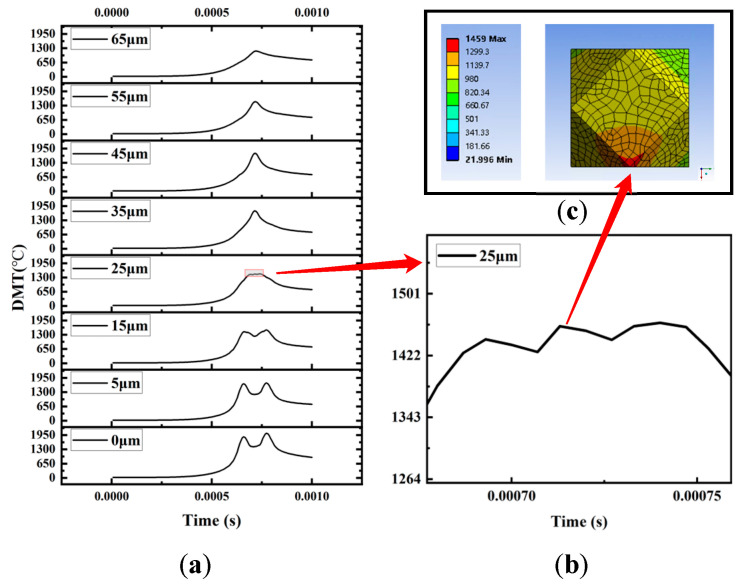
(**a**) The DMT curves of 0μm~55 μm laser scan path offset (**b**) The DMT curves of 25 μm laser scan path offset local enlarged view (**c**) The temperature distribution cloud of 25 μm laser scan path offset at 7.12 × 10^−4^ s.

As shown in Figure 11a, by adjusting the offset distance between the laser scan path and the diamond, the DMT curves corresponding to different offsets can be obtained. Within the offset range of 0 μm−15 μm, the DMT curve exhibits a pronounced “double-peak” structure. When the offset reaches 25 μm, the DMT curve undergoes a change, and a small peak emerges between the original two peaks. The height of this newly appeared peak tends to be between the original two peaks, as shown in Figure 11b. With the laser scan offset, the DMT curve becomes a single peak. The reason for this situation is that in the process of laser irradiating a diamond, with the offset of the laser scanning path, the area of the laser directly irradiating the diamond decreases, and the area of the laser directly irradiating the CuSn10 powder increases, resulting in the valley between the two peaks gradually being filled and developing into a small peak. Eventually, the small peak replaced the double peak and evolved into a single peak. Figure 11c shows the temperature distribution cloud corresponding to the small peak at the offset of 25 μm. The maximum temperature of a diamond occurred at the edge of the diamond, indicating that the temperature change of the small peak is caused by the heat transfer of the CuSn10 powder near the diamond.

**Figure 12:**
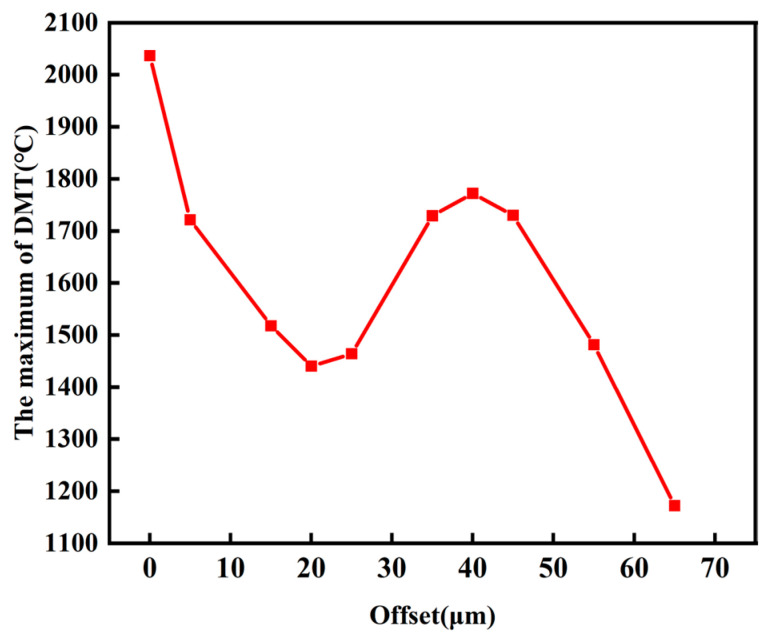
Offset versus the diamond temperature curve and the schematic of the relative position of the spot.

Figure 12 shows the offset versus the maximum DMT. The maximum DMT means the maximum diamond temperature during the whole process. It can be seen that the maximum DMT does not monotonically decrease as the laser scanning path deviates. This can be explained by the fact that when the center of the laser spot is in proximity to the edge of the diamond, a larger area of the CuSn10 is irradiated by the spot. Thus, the molten pool absorbs more energy, resulting in a higher temperature of the diamond. On the other hand, with the continuous offset of the laser scan path, although the molten pool absorbs more energy, the diamond is not in the center of the molten pool. Therefore, the molten pool energy cannot be transferred to the diamond in time, resulting in a continuous decline in diamond temperature.

Due to the effect of gravitational forces, Marangoni effects, and laser irradiation, the migration of diamond particles within the molten pool becomes an inescapable phenomenon, exhibiting distinct directional tendencies [18]. The spatial relationship between diamond particles and the molten pool or the laser path stands out as a pivotal determinant influencing diamond temperature, a parameter of utmost significance in orchestrating effective mitigation of diamond graphitization and the systematic positioning of diamond particles.

### 4.3. Effect of Diamond Particle Size on Diamond Temperature

**Figure 13:**
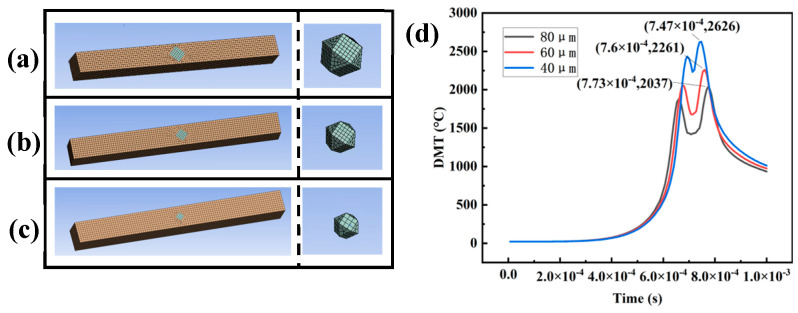
Simulation model of different diamond diameter (**a**) 80 μm (**b**) 60 μm (**c**) 40 μm (**d**) Temperature variation curves of 80 μm, 60 μm and 40 μm diamond.

Figure 13 presents a simulation model showcasing different diamond diameters, namely (a) 80 μm, (b) 60 μm, and (c) 40 μm. In this model, the diamond is embedded in the surface of the powder bed. Figure 13d displays the DMT curves of diamonds with diameters of 80 μm, 60 μm, and 40 μm, without laser scan path offset. Notably, even after altering the diamond particle size, the DMT curves continue to exhibit a “double-peak” structure. Moreover, as the diamond diameter decreases, the diamond temperature rises.

Diamond exhibits a low laser absorption rate, resulting in less laser energy being absorbed during the LPBF process. Consequently, a larger diamond absorbs less laser energy. According to

(5) $$\Delta T=\frac{Q}{\rho vc},$$

where *c* is the specific heat capacity, *Q* is the absorbed heat, *ρ* is density, *v* is volume, and *T* is temperature variation. The temperature of the larger diamond changes more slowly. At the same laser scanning time, the temperature of the larger diamond is lower than that of the smaller diamond. Furthermore, a diamond possesses high thermal conductivity, allowing it to serve as a micro-channel for heat dissipation throughout the entire structure, and diamond particles enhance the heat dissipation effect. As the diamond diameter decreases, the two peaks of the DMT curve become closer to each other due to the reduced duration of diamond irradiation.

**Figure 14:**
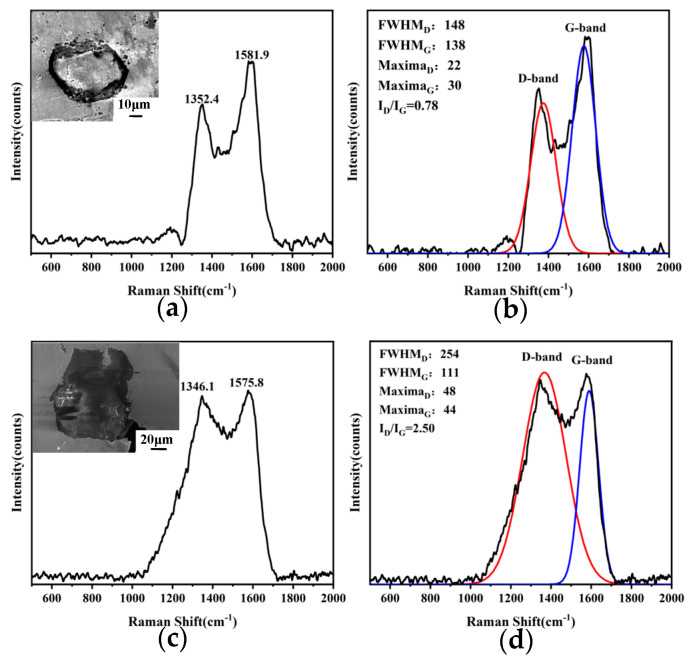
(**a**,**b**) 45 μm−53 μm and (**c**,**d**) 74 μm−90 μm diamond diameters SEM image and Raman spectra.

The thermal damage of diamond particles in the composites was analyzed using Raman spectroscopy, as depicted in Figure 14. The Raman test results of the diamond without graphitization will show a long and sharp characteristic peak at 1332 cm^−1^. The graphitized diamond will have a characteristic peak near 1580 cm^−1^. It is worth noting that the samples in Figure 8 and Figure 14 used the same sample image. During the process of thermal damage or amorphous carbon formation, there is a decline in SP_3_ hybridization and an increase in SP_2_ hybridization. This phenomenon is reflected in the Raman spectra by a reduction in peak intensity near 1332 cm^−1^ and an augmentation in peak intensity near 1580 cm^−1^. Figure 14 further demonstrates that, under the same parameters, diamonds with diameters ranging from 45 μm−53 μm and 74 μm−90 μm exhibit varying degrees of thermal damage. Notably, as the diamond particle size increases, the intensity ratio of the diamond-phase peak to the graphite-phase peak (I_D_/I_G)_ increases. This observation indicates a decrease in the degree of thermal damage as the diamond particle size expands.

Furthermore, Figure 13 illustrates that as the diameter of the diamond decreases, the two peaks in the DMT curve become closer together, resulting in a reduced temperature variation. This phenomenon has the advantage of alleviating the self-induced stress within the diamond, thereby mitigating issues such as diamond cracking, splashing, and thermal damage caused by excessive stress. Therefore, despite diamonds with smaller diameters exhibiting higher temperatures under identical laser process parameters, they effectively minimize the self-induced stress and mitigate the risk of diamond damage. Through further fine-tuning of the laser process parameters, diamonds with smaller diameters are deemed more suitable for laser powder bed fusion applications. Sun et al. proposed that residual stress is the result of the joint action of thermal stress and volume stress, and the increase of volume will lead to the increase of volume stress [19]. The difference in thermal expansion coefficient between diamond and matrix metal is the main factor to produces residual stress. When the thermal stress is the same, the volume change of the larger diamond is more obvious, and the volume stress is greater.

### 4.4. Effect of Coating on Diamond Temperature

Based on the “double-peak” model, it has been concluded that the dominant factor affecting the DMT curve is the heat transfer from the molten pool. In order to resist the temperature of the molten pool, the surface of the diamond can be coated with a metal coating of titanium with low thermal conductivity.

**Figure 15:**
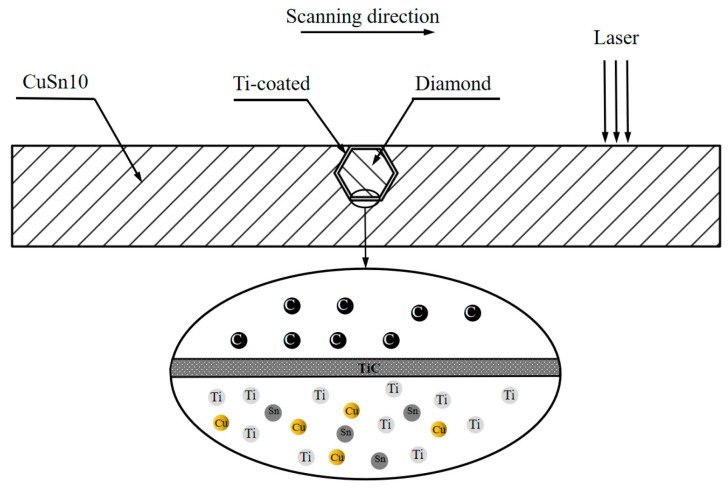
Schematic of the CuSn10 Ti-coated diamond metal matrix diamond composites by LPBF.

Figure 15 illustrates the CuSn10 Ti−coated diamond metal matrix diamond composites by the LPBF process. The Ti coating serves several purposes: it enhances the infiltration of the diamond into the substrate, allowing for a greater burial depth; it promotes diffusion fusion with the substrate, thereby strengthening the surrounding region of the diamond; and it reacts with the diamond to form TiC, facilitating a metallurgical bond between the substrate and the diamond. This bonding mechanism significantly improves the adhesion of the matrix to the diamond [20]. Furthermore, the coating provides protection for the diamond, effectively enhancing its oxidation resistance and high-temperature resistance, while also inhibiting the SP_3_ → SP_2_ phase transition of the diamond at elevated temperatures [21,22]. However, it should be noted that the high absorption rate of the coated metal results in increased laser energy input to the diamond. Additionally, the thermal conductivity of the coated metal is significantly lower than that of the diamond, hindering heat exchange between the diamond and its surroundings and reducing the diamond’s heat dissipation efficiency. Therefore, the temperature of the diamond rises, posing a risk of thermal damage.

**Figure 16:**
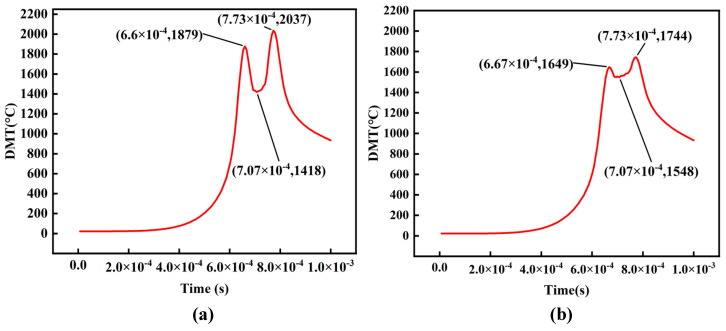
(**a**) Bare diamond temperature variation curve (**b**) Ti−coated diamond temperature variation curve.

As shown in Figure 16, the temperature of the Ti-coated diamond reaches 1744 °C, which is lower than the temperature of the bare diamond (2037 °C) under the same process parameters. This finding serves as evidence that the coating effectively prevents the transfer of heat from the molten pool to the diamond. However, the observed increase in the valley temperature indicates that the coating influences the heat dissipation performance of the diamond.

**Figure 17:**
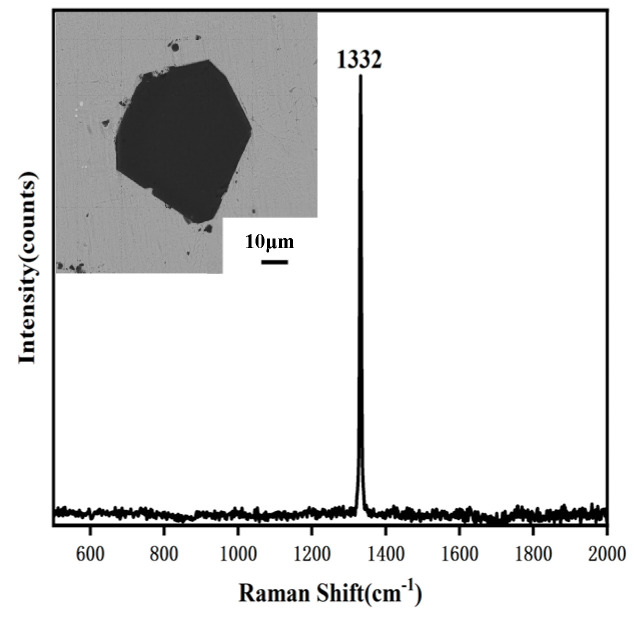
SEM image and Raman spectral of Ti−coated diamond.

To investigate the extent to which the Ti coating affects the thermal damage to the diamond, SEM and Raman analyses were conducted on the processed Ti-coated diamond particles. Figure 17 illustrates the results, which reveal that the Ti-coated diamond retains its original diamond morphology without any signs of thermal damage. The Raman spectrum analysis demonstrates that the characteristic peak of the diamond at 1332 cm^−1^ is sharp and elongated, indicating the preservation of a well-defined crystalline structure and the absence of thermal damage. This observation is in stark contrast to the experimental results obtained with uncoated diamond in Section 4.3, further confirming that the coating effectively shields the diamond, thereby maintaining its morphology and lattice properties. The results of this experiment are confirmed in the literature [23]. Ti−coated diamond composite maintained a complete diamond crystal shape, and the interface between diamond and matrix was flat and clear. The Ti −coated not only has a good reaction with the diamond, but it also diffuses with the matrix to improve the bonding interface strength. Therefore, the coated diamond is better equipped to exhibit its intrinsic characteristics.

## 5. Discussion

While the preceding experiments have elucidated the temperature characteristics and graphitization mechanisms of diamonds, it is imperative to acknowledge that they have not undergone a comprehensive and rigorously precise verification process to substantiate the conclusions drawn. To enhance the accuracy and reliability of the temperature model and numerical simulation results presented in this paper, it is imperative to employ more refined temperature measurement devices and conduct supplementary experimental investigations.

To this end, the implementation of a highly sensitive infrared temperature measurement system encompassing the entire LPBF process, coupled with machine vision analysis for precise assessment of diamond particle positioning on the surface, is paramount in achieving an accurate measurement of diamond temperatures.

Furthermore, process refinement has enabled the fabrication of CuSn10-Diamond composite materials with varying diameters, ensuring the absence of damage. The assessment of diamond residual stress can be accomplished through the measurement of the 1332 cm^−1^ Raman peak shift [24].

It is worth noting that the aforementioned experimental endeavors will be continued and further expanded upon in subsequent phases of this research initiative.

## 6. Conclusions

In this paper, a finite element simulation model was developed by the powder absorption theory. This model shows the temperature behavior of the diamond during the LPBF process. The temporal evolution of diamond temperature was examined and experimental analyses to unravel the intricate graphitization mechanisms that govern a diamond’s behavior were conducted. Furthermore, leveraging this model, the multifaceted influence of various parameters, specifically the laser scanning path’s relative positioning with respect to the diamond, diamond diameter, and the utilization of Ti-coated diamond particles was explored. From our investigations, several key findings have emerged:

(1) The simulation results suggest a double-peak structure in the diamond temperature. This phenomenon underscores the predominant heat transfer mechanism governing diamonds within LPBF, with the primary source of diamond heating originating from the molten pool’s thermal influence. In contrast, the direct laser irradiation of diamonds emerges as a secondary factor affecting diamond temperature.

(2) Experimental observations have underscored that diamond defects impede efficient heat transfer, resulting in temperature accumulation at these defects. The temperature accumulation leads to a rise in diamond temperature, ultimately culminating in the phase transition toward graphitization.

(3) By manipulating the relative positioning of the laser scanning path in relation to the diamond and varying the size of diamond particles, the fundamental control over the “double-peak” structure was achieved. Altering the offset of the laser scanning path relative to the diamond induces changes in both the peak temperature and the temperature structure. This empirical evidence substantiates the significant influence of the laser scanning path and the Marangoni effect on diamond temperature. Additionally, reducing the diameter of the diamond particles has been demonstrated to diminish the peak separation, increase the valley depth between the double peaks, render the temperature curve smoother, and consequently reduce the residual stress experienced by the diamond.

(4) The Ti coating with low thermal conductivity effectively prevents the heat transfer from the molten pool to the diamond and reduces the thermal damage of the diamond.

## Figures and Tables

**Table 1:** LPBF process parameters for diamond composites.

Process Parameters	Numerical Value
Laser power (*P*)/W	180
Scanning speed (*v*)/mm/s	700
Scan pitch/mm	0.05
Spot diameter/mm	0.05
Layer thickness/mm	0.07

**Table 2:** The thermophysical parameters of the CuSn10 and diamond.

	Density kg/m^−3^	Melting Temperature °C	Specific Heat J/(kg°C)	Thermal Conductivity W/m°C	Absorptivity
CuSn10	8780	999	Figure 4a	Figure 4b	38% [15]
Diamond (powder)	3510	/	472	2000	25% [12]
Titanium	4500	1668	522	21	26% [16]

## Data Availability

Data sharing is not applicable to this article.
